# Characterization of a Novel Putative S-Adenosylmethionine Decarboxylase-Like Protein from *Leishmania donovani*

**DOI:** 10.1371/journal.pone.0065912

**Published:** 2013-06-19

**Authors:** Saurabh Pratap Singh, Pragati Agnihotri, J. Venkatesh Pratap

**Affiliations:** Molecular & Structural Biology Division, Central Drug Research Institute, Chattar Manzil, Mahatma Gandhi Marg, Lucknow, Uttar Pradesh, India; Technion-Israel Institute of Technology, Israel

## Abstract

In addition to the S-adenosylmethionine decarboxylase (AD) present in all organisms, trypanosomatids including *Leishmania spp.* possess an additional copy, annotated as the putative S-adenosylmethionine decarboxylase-like proenzyme (ADL). Phylogenetic analysis confirms that ADL is unique to trypanosomatids and has several unique features such as lack of autocatalytic cleavage and a distinct evolutionary lineage, even from trypanosomatid ADs. In *Trypanosoma* ADL was found to be enzymaticaly dead but plays an essential regulatory role by forming a heterodimer complex with AD. However, no structural or functional information is available about ADL from *Leishmania spp*. Here, in this study, we report the cloning, expression, purification, structural and functional characterization of *Leishmania donovani* (*L. donovani*) ADL using biophysical, biochemical and computational techniques. Biophysical studies show that, *L. donovani* ADL binds S-adenosylmethionine (SAM) and putrescine which are natural substrates of AD. Computational modeling and docking studies showed that in comparison to the ADs of other organisms including human, residues involved in putrescine binding are partially conserved while the SAM binding residues are significantly different. *In silico* protein-protein interaction study reveals that *L. donovani* ADL can interact with AD. These results indicate that *L. donovani* ADL posses a novel substrate binding property and may play an essential role in polyamine biosynthesis with a different mode of function from known proteins of the S-adenosylmethionine decarboxylase super family.

## Introduction

Visceral Leishmaniasis or kala-azar is a one of the most neglected diseases caused by the parasitic protozoan *Leishmania*. It causes an estimated 500,000 new cases of disease and more than 50,000 deaths every year with 90% occurring in Bangladesh, Nepal, India, Sudan, Ethiopia and Brazil [1]. The disease is becoming a cause of concern with the advent of HIV-leishmaniasis co-infection [2]–[5]. Without treatment, visceral leishmaniasis is always fatal. The available drugs have limitations like toxicity, difficult dosing regimens, emerging resistance and therefore new drugs are required. The first step in a rational drug design approach is the identification, structural and functional characterization of proteins in pathways that are indispensable to the pathogen and are sufficiently distinct from their human homologues. The polyamine biosynthesis pathway in *Leishmania* can be one such pathway [6]–[9]. Polyamines such as- putrescine, spermidine and spermine are essential components of the cell involved in cell growth, differentiation and proliferation. One of the two drugs certified by the US Food and Drug Administration for the treatment of late stage African sleeping sickness caused by *Trypanosoma brucei* (*T. brucei*) is eflorinithine, a suicide inhibitor of ornithine decarboxylase (ODC), an enzyme in the polyamine biosynthesis pathway [10], further validate the importance of this pathway. In eukaryotes including *Leishmania*, putrescine is synthesized from L-ornithine through a decarboxylation reaction catalyzed by ODC. Subsequently, spermidine is synthesized by the incorporation of an aminopropyl group in a reaction catalyzed by the enzyme spermidine synthase. The aminopropyl group is provided by decarboxylated S-adenosylmethionine, which is produced in a reaction catalyzed by S-adenosylmethionine decarboxylase referred here as AD. Spermidine is subsequently conjugated with glutathione to synthesize a unique polyamine in trypanosomatids i.e. trypanothione, which is essential for cellular redox reactions and nucleotide synthesis [11].

AD (E.C 4.1.1.50) belongs to a small group of enzymes that depend on a pyruvoyl cofactor for decarboxylation reaction. AD is expressed as an inactive proenzyme that undergoes an autoprocessing reaction in humans, *Trypanosoma* and plants [12]–[15]. Autoprocessing involves an internal serinolysis reaction leading to the cleavage of the proenzyme backbone into two subunits, a small β subunit and a large α subunit and a. The catalytic mechanism involves the generation of a pyruvoyl group at the N-terminus of the α chain and requires the amino acid sequence ES as its cleavage site. The substrate S-adenosylmethionine (SAM) binds to this pyruvoyl group through a Schiff base and the decarboxylation reaction proceeds with the transfer of a pair of electrons from the substrate to pyruvoyl group [16]–[18]. Autoprocessing as well as decarboxylation are stimulated by putrescine in humans [19], [20] but not in plants and they lack the putrescine binding site [21]. In *T. brucei* putrescine is not necessary for the autoprocessing reaction but it stimulates the decarboxylation reaction, although the decarboxylation reaction is not as efficient as found in human and plant AD [12], [22]–[25]. The structural details of the enzymes belonging to the AD super family mainly come from crystal structures of human and potato ADs [19], [21]. Although these two proteins differ in their oligomeric association, the human AD exist as a dimer while the potato AD is monomeric, their crystal structures reveal an identical fold comprising of a four layer αββα sandwich. Each central β sheet comprises of eight antiparallel β strands, flanked by α helices on either side. The six α helices observed in the monomer are all amphipathic, packed tightly against the outer faces of the β sandwich of AD. However, no structure of AD from any trypanosomatids has been reported till date.

Apart from AD, trypanosomatids including *Leishmania* possess another AD like protein annotated in databases as a putative S-adenosylmethionine decarboxylase-like proenzyme, here onwards referred as ADL (NCBI gene accession no. CBZ36337.1). The *T. brucei* ADL was shown to be paralogous to AD, did not undergo autocatalysis and could not retain its native confirmation in the absence of AD. *T. brucei* ADL was also found to be enzymatically dead, but playing a significant role as a regulatory subunit by forming a high affinity heterodimer with AD, upregulating the enzyme activity by ∼1200 fold [26]. Though ADL controls the activity of AD in trypanosomatids, no information is known regarding the mechanism of regulation or the substrate binding aspect of ADL. ADL is proposed to have evolved through duplication of the ancestral main AD and subsequent mutational drift that lead to the loss of its catalytic activity but retaining its allosteric regulatory function. The conditional knockouts of *T. brucei* AD and ADL revealed that depletion of either protein led to a reduction in the level of spermidine and trypanothione ultimately resulting in the death of the parasite. This implies that ADL is an essential protein for the survival of *Trypanosoma* [26], [27]. Comparison of the pairwise amino acid sequence alignments of *L. donovani* AD and ADL with human AD suggest that the ADL has a significantly lower sequence identity (∼13%) than *L. donovani* AD (∼25%). Further, *L. donovani* AD shares six residues which are involved in SAM positioning and binding and two among the three putrescine binding residues with the human AD. This indicates that in comparison to AD, ADL may be a better target for any rational drug development approach. Here, in this study we report the cloning, expression, purification, structural and functional characterization of *L. donovani* ADL, to ascertain its viability in drug development.

## Materials and Methods

### Sequence and phylogenetic analysis

The amino acid sequences of ADs and ADLs of kinetoplastida, such as *L. major, L. infantum, L. donovani, L. brazilensis T. brucei* and *T. cruzi* were retrieved from the Swiss-Prot gene database and domain architecture was predicted using CDD (http://www.ncbi.nlm.nih.gov/Structure/cdd/cdd.shtml) [28] and the fold analyzed by FoldIndex (http://bip.weizmann.ac.il/fldbin/findex) [29]. The phylogenetic tree was drawn with the help of MEGA 5.0 [30] by using the neighbor joining method based on the bottom-up clustering algorithm [31]. The sequences were aligned using ClustalW version 1.8 [32]. The secondary structure was predicted by using PHD server (http://npsapbil.ibcp.fr) [33]. The Q site finder (http://www.modelling.leeds.ac.uk/qsitefinder/) was used to identify conserved functional residues [34].

### Cloning, protein expression and purification

The 894 bp long *L. donovani* ADL gene was amplified from the genomic DNA of *L. donovani* using specific sense “5ATTGGATCCATGTCGCTGTGGGGAGGGTTTTCGAACCC3” and anti-sense “5GGGAAGCTTATCTGCACTGCGGGCGAATAGTAGTTGGTG3” primers designed using Oligo software, with sites for BamHI and HindIII restriction enzymes (underlined) respectively. The amplified gene was cloned in T/A vector pTZ57R/T (InsTA cloneTM PCR cloning kit, Fermentas International Inc.) and then sub-cloned downstream of the T5 promoter expression vector pQE30 (Qiagen) between BamHI and HindIII sites. *E. coli* TG1 host cell was transformed with the recombinant plasmid pQE30-ADL and used for over-expression.

Luria-Bertani (LB) broth containing ampicillin (100 µg/ml) was inoculated with the *E. coli* containing the pQE30-ADL plasmid and cultured overnight at 37°C. Fresh LB broth containing ampicillin (100 µg/ml) was inoculated with 1∶100 dilution of this seed culture and incubated at 37°C to an OD_600_ of ∼0.6. Over-expression was then induced by adding 1 mM isopropyl-1-thio-β-galactopyranoside (IPTG) and allowed to grow overnight at the same temperature. The culture was subsequently harvested by centrifugation, resuspended in a buffer containing 50 mM Tris-HCl pH 7.0 and 200 mM NaCl. Cells were lysed by sonication with a 20 second on and 15 second off pulse for 30 minutes. Cell debris was removed by centrifugation at 13,000 rpm for 30 minutes and the supernatant loaded on an IMAC column pre-equilibrated with the same buffer. The column was incubated for an hour and subsequently washed with buffers containing 10 mM and 60 mM imidazole. Protein was then eluted with 300 mM imidazole in the same buffer. The eluted protein was dialyzed overnight into 50 mM Tris-HCl pH 7.0, 50 mM NaCl, 1 mM putrescine and 3 mM β-me, concentrated using 10 kDa cutoff centricon (Amicon) and loaded on size exclusion chromatography for the second step purification and oligomerization analysis.

### Size exclusion chromatography

Size exclusion chromatography was performed on the Superdex™ 75 10/300 prepacked column (manufacturer's exclusion limit 70 kDa for proteins) on an ΔKTA-FPLC (GE HealthCare Biosciences). The dialyzed and concentrated ADL protein (1 mg/ml) was loaded onto the column pre-equilibrated with a buffer containing 50 mM Tris-HCl pH 7.0, 50 mM NaCl, and 3 mM β- me. The elution was carried out isocratically at a flow rate of 0.3–0.4 ml/min and monitored using the absorbance at 280 nm. All measurements were made at 25°C. Calibration of the column was performed using the low molecular weight standard kit (GE Healthcare) containing conalbumin, ovalbumin, carbonic anhydrase, RNAase and aprotinine as reference proteins.

### Determination of protein concentration

The protein concentration was determined by using Bradford method [35]. The standard curve was plotted with bovine serum albumin in range of 0–22 mg/ml.

### Limited proteolysis

Trypsin digestion was used to analyze the domain architecture of ADL protein. Trypsin was added to ADL in a 200∶1 ratio and reactions were set up for 10 min, 30 min, 1 hour and overnight and, stopped by adding 1 mM PMSF. The effect of substrates such as SAM and putrescine on limited proteolysis was observed in the concentration range 0–80 µM and 0–90 µM respectively in an overnight reaction. All samples were analyzed on a 12% SDS-PAGE.

### Fluorescence measurements

The fluorescence emission spectra using intrinsic (Tryptophan) and extrinsic (1-anilinonapthelene-8-sulfonate, ANS) fluorophores were recorded on a Perkin Elmer LS50b luminescence spectrometer at 25°C. Cuvettes with 5 mm path length were used and 2 µM ADL in 50 mM Tris-HCl pH 7.0, 50 mM NaCl and 3 mM β-me was used for these studies. For tryptophan fluorescence, the protein sample was excited at 295 nm and the emission spectra recorded in the range 300–400 nm. For ANS binding studies, the corresponding values were 370 nm and 400–600 nm respectively. For ANS binding studies, a dye to protein molar ratio of 20∶1 was used and the samples were incubated with ANS for 30 minutes and gently shaken before taking measurements. The effect of urea and Guanidinium chloride (GdmCl) on ADL was observed in the concentration range 0–6 M by using tryptophan and ANS fluorescence. Fluorescence spectra with increasing concentrations of SAM and putrescine (0–200 µM) up to saturation were measured and the change in tryptophan fluorescence observed at 341 nm. As a control, titrations with buffer alone did not produce any significant change in the emission signal. The change in fluorescence can be then related to the binding of SAM and putrescine by the following equation [36], [37].
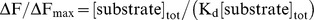



Where ΔF is the magnitude of the difference between the observed fluorescence intensities in the presence and absence of the substrate at a given concentration of substrate, ΔF_max_ is the difference between the observed fluorescence intensities at zero and saturating substrate concentration, [Substrate] _tot_, and K_f_ is the apparent dissociation constant. The K_d_ values were determined from a non-linear least-squares regression analysis of titration data. With all samples, fluorescence spectra were corrected for the background fluorescence of the solution (buffer+substrate). Deconvolution of curves was performed using the Prism software (GraphPad software Inc) & Phase diagrams describing GdmCl and urea induced changes of fluorescence intensities were constructed.

### Circular dichroism measurements

The far-UV CD measurements were made on a Jasco J810 spectropolarimeter and Chirascan™ CD spectropolarimeter (Applied Photophysics) calibrated with ammonium (+)-10- camphorsulfonate. Three spectra (200–260 nm, scan-speed 10 nm/min) from 2 µM protein samples in 50 mM Tris– HCl pH 7.0, 50 mM NaCl were taken and averaged. All measurements were taken using standard protocol [38]. The K2D3 software (http://www.ogic.ca/projects/k2d3/) was used to estimate the secondary structure content of the protein [39]. The effect of SAM and putrescine was observed in the concentration range 0–200 µM. Secondary structure was observed in a buffer containing 50 mM NaCl and 3 mM β-me with pH profile varying from 4.0 to 9.0 i.e. 50 mM sodium acetate pH 4.0, 50 mM MES pH 6.0, and 50 mM Tris-HCl pH 7.0 and 9.0. The thermal denaturation experiments of *L donovani* ADL, in apo and in complex with SAM and putrescine were performed in the same spectropolarimeter using the standard protocol between 25°C–90°C. The folded fraction of the protein at these temperature values were determined [40].

### Homology modeling and docking studies of *L donovani* ADL

In the absence of suitable template hits by PSI-BLAST against the PDB, the templates for homology modeling were found by searching structures with similar fold, using the PHYRE server (http://www.sbg.bio.ic.ac.uk/~phyre/) which too takes the amino acid sequence as input and combines predicted secondary structure information in addition to PSI-BLAST generated alignment profile [41]. PHYRE identified four structures in the PDB as potential templates with 100% confidence: three human AD structures (in apo and liganded forms, PDB IDs 1I7B, 1MSV, 1JLO) and the structure of potato AD (PDB ID 1MHM). The human structure was taken as the template for homology modelling. Homology modeling was performed using MODELLER 9.10 using default parameters with the pairwise sequence alignment file of the target (*L. donovani* ADL) and the template as input [42]. Five models were obtained as modeller output with each template and were ranked on the basis of their minimum internal energy. The model with minimum internal energy and root mean square deviation from the template was used for further validation. The quality of these models were validated using MolProbity and PROCHECK [43], [44]. Homology models of AD and ADL from *L. major, L. infantum, L. donovani and L. brazilensis* were also made to confirm the interactions involved in heterodimer formation.

Molecular docking study was performed using docking software AUTODOCK 3.0 with default parameters [45]. The *L. donovani* ADL model was docked with the substrates SAM and putrescine. Ten conformations of each ligand were obtained and ranked according to their minimum docking energies. The best conformation selected on the basis of minimum docking energy, and no steric clashes between the residues involved in binding. The conformation which obeys these conditions was used for the active site analysis. The protein-ligands interaction diagram was generated by using LIGPLOT tool [46].

The protein-protein interaction was analyzed by STRING 9.0 (http://string-db.org) [47]. STRING 9.0 is an interacting genes database which requires gene ID or amino-acid sequence as input and predicts interaction on the basis of genomic context, high-throughput experiments, co-expression, experiments and previous knowledge. Protein-protein docking was carried out independently using two different servers, ClusPro 2.0 (http://cluspro.bu.edu/login.php) and GRAMM-X (http://vakser.bioinformatics.ku.edu/resources/gramm/grammx) [48], [49]. While ClusPro 2.0 utilizes the models or PDB IDs of query interacting partners as input and performs rigid body docking to give a docked model, GRAMM-X is based on a Fast Fourier transform algorithm utilizing shape complementarity and a softend-Lennard-Jones potential function.

## Results and Discussion

### Sequence analysis

**Figure 1:**
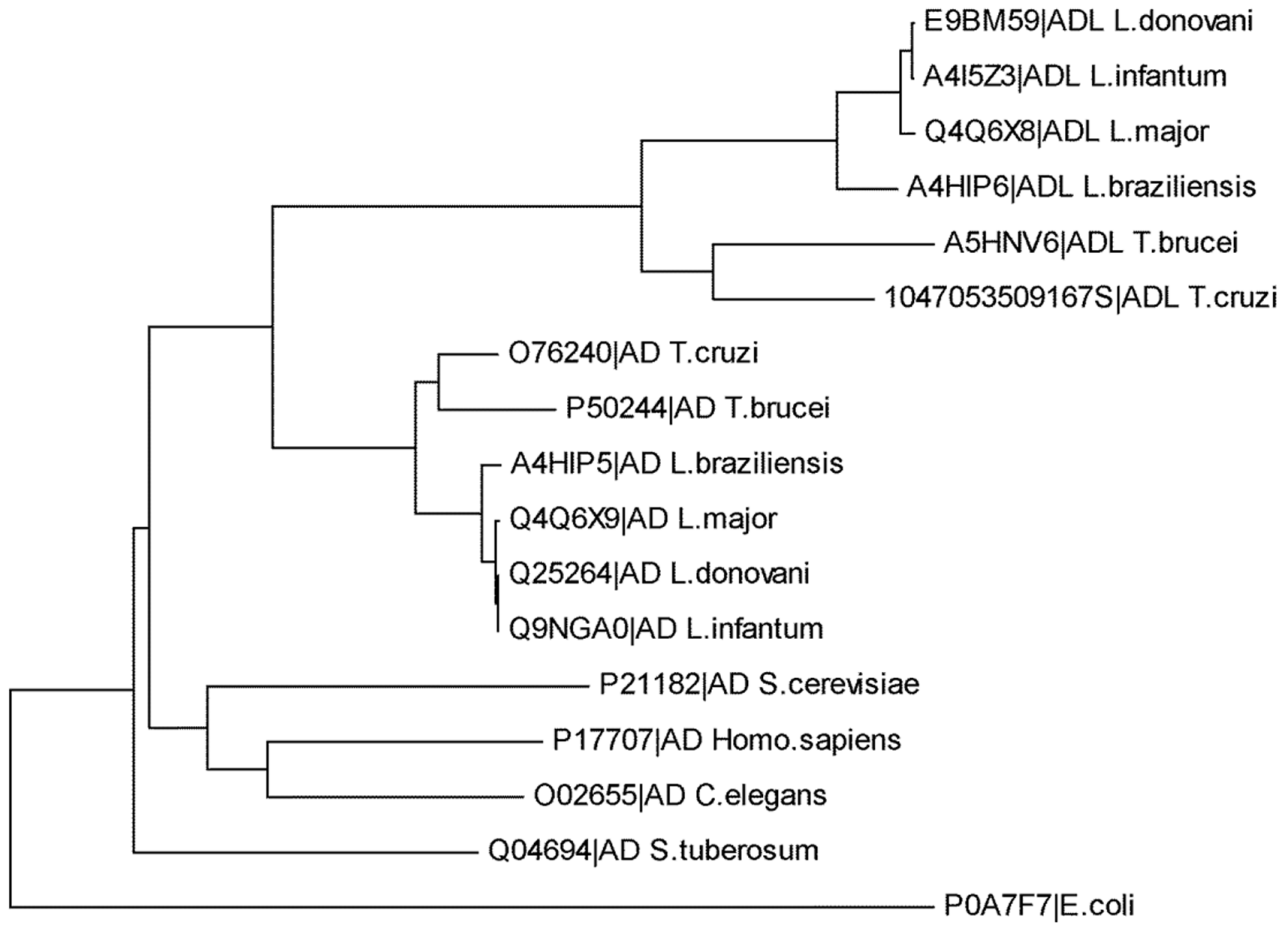
Phylogenetic analysis showing evolutionary patterns of AD and ADL in trypanosomatids. Amino acid sequences were retrieved from the Swiss-Prot gene data base and phylogenetic tree was constructed using MEGA 5.0 software showing the different clusters of AD and ADL.

ADL belongs to the S-adenosylmethionine decarboxylase superfamily, having a single domain as predicted by the conserved domain prediction and FoldIndex respectively, while a BLAST search against the non redundant protein database shows that ADL is found only in trypanosomatids. PSI-BLAST against the PDB did not show any significant hits. Phylogenetic analysis of the amino acid sequences of AD and ADL (Figure 1) shows that trypanosomatids have developed two copies of AD, after their divergence from other eukaryotes. Willert *et. al.*, [26] have suggested that the most likely reason for the presence of two copies of AD in *trypanosoma* lies in the regulation of production of polyamines in a dynamic way, by regulating the expression level of ADL under different environmental conditions. *L. donovani* being a member of the same kinetoplastid family, might also have evolved these two copies for the same reason. *Leishmania* ADLs have developed at a later stage as compared to ADs. Analysis also suggests that the *L. donovani* ADL is evolutionarily more distant from the human AD than *L. donovani* AD.

**Figure 2:**
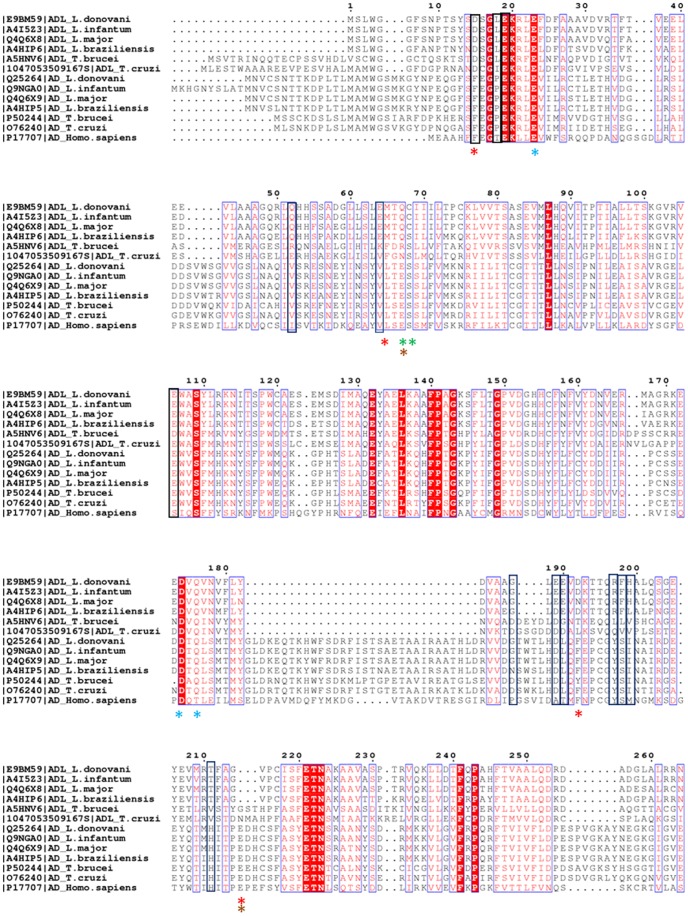
Multiple sequence alignment of the amino acid sequences of AD and ADLs of *L.* donovani, L. infantum, L. major, L. brazilensis, T. brucei and T. cruzi and human AD. Based on this alignment, the residues of human AD involved in autocatalysis are shown by a green asterisk; SAM positioning by red asterisk; SAM binding by brown asterisk and putrescine binding shown in cyan asterisk. Molecular docking studies of SAM and putrescine with homology model of L. *donovani* ADL suggest that SAM binding residues are not conserved while putrescine binding residues are found partially conserved. The residue comprising SAM binding pocket and involved in H-bond interaction with SAM in *L. donovani* ADL are enclosed in blue boxes. Putrescine binding residues of *L. donovani* ADL are represented by black boxes. Alignment is made with the help of Espript 2 [54].

Multiple sequence alignment of ADs from human, *Leishmania spp*. and *Trypanosoma spp.* along with ADLs from *L. donovani*, *L. major L. infantum L. brazilensis, T. brucei* and *T. cruzi* was done to identify conserved & functionally important residues (Figure 2). The alignment shows that most of the functionally important residues are found to be identical between the human and trypanosomatids ADs while the trypanosomatid ADLs have significantly different residues (Figure 2). Glu 67 and Ser 68, residues essential for autocatalysis in human AD [18] are also conserved in trypanosomatids ADs which too undergoes autocatalysis [9], [22]. In the case of trypanosomatids ADLs including *L. donovani*, these residues are not conserved, suggesting the absence of autocatalysis reaction, as seen in *T. brucei* ADL. The absence of autocatalysis suggests that ADL from trypanosomatids should not be annotated as proenzyme in the gene database. As autocatalysis is an essential step of decarboxylation mechanism, it also suggests that the trypanosomatids ADLs are probably not capable of SAM decarboxylation [26].

Structure-based sequence analysis of the SAM and putrescine bound crystal structure of human AD (PDB ID 1I7B) [50] shows that the residues involved in SAM positioning and binding i.e., Phe7, Leu65, Glu67, Phe223, Glu247 are conserved in *L. donovani* AD (Phe32, Leu87, Glu89, Phe248, Glu271 respectively), and interestingly, none of these residues are conserved in *L. donovani* ADL. In fact, a majority of them are chemically different in *L. donovani* ADL- Phenylalanines 7 and 223 correspond to Asp 15 and 192 in ADL, the stretch of residues corresponding to human Glu 247 is deleted in and at position 65, *L. donovani* ADL has a methionine instead of a leucine. Residues involved in putrescine binding, though are partially conserved in *L. donovani* ADL as well. In summary, the residues involved in autocatalysis and SAM positioning and binding are entirely different which suggests that the *L. donovani* ADL might function in a novel manner. To further explore and understand the role of *L. donovani* ADL, the protein was cloned, over-expressed, purified and structurally and functionally characterized.

### Cloning and purification of *L. donovani* ADL

**Figure 3:**
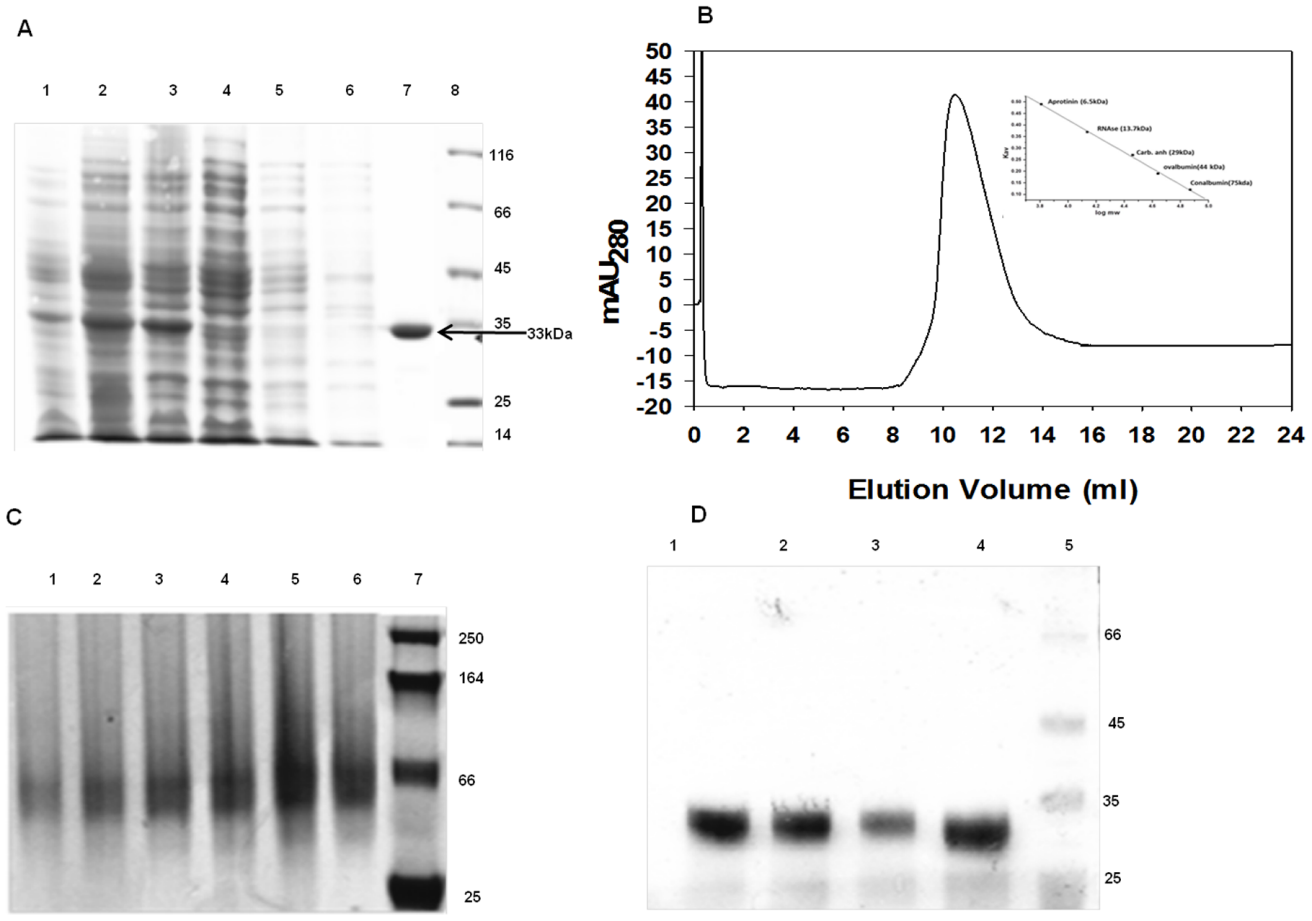
Over-expression, purification, size exclusion chromatography and trypsin digestion of of *L.* *donovani* ADL. (A) 12% SDS PAGE of the purified *L. donovani* ADL. M-unstained protein marker; lane 1-unduced; lane 2-induced lane 3-load; lane 4-flow through; lane 5-equilibration; lane 6-wash; lane 7-elution of *L. donovani* ADL showing single band after metal affinity chromatography. (B) Size exclusion chromatography profile of *L. donovani* ADL showing protein elution at 10.4 ml on a Superdex 75 column, corresponding to 66 kDa molecular weight i.e. dimer. (C) Confirmation of the dimer by Native Polyacrylamide Gel Electrophoresis. (D) Trypsin assisted limited proteolysis analysis of *L. donovani* ADL at different time intervals showing that trypsin has no effect on *L. donovani* ADL.

The *L. donovani* ADL gene was cloned, over-expressed and purified using IMAC and was observed on SDS-PAGE as a single band corresponding to 33 kDa, indicating the absence of any autocatalytic cleavage reaction (Figure 3A), consistent with the sequence alignment result (Figure 2). *L. donovani* ADL after IMAC purification was found to undergo irreversible precipitation even after dialysis and the rate of precipitation was directly proportional to the protein concentration. Incorporation of putrescine (1–5 mM) in the buffer at every step of purification or keeping protein concentration below 0.2 mg/ml decreased the precipitation, suggesting the stabilizing effect of putrescine.

### Biophysical characterization of *L. donovani* ADL

Size exclusion chromatography profile of *L. donovani* ADL shows that it elutes at 10.4 ml on the Superdex™ 75 10/300 column, corresponding to ∼66 kDa, suggesting *L. donovani* ADL is a dimer (Figure 3B). The dimer was further confirmed by native PAGE (Figure 3C) and is consistent with other trypanosomatid and human ADs. However, it does not show any aggregation, unlike *T. brucei* ADL which partially aggregates in the absence of AD [26].

Limited proteolysis experiment with trypsin failed to show any additional bands in spite of the sequence having 25 Lys and Arg residues, suggesting that the whole protein adopts a single folded structure (Figure 3D) with the positively charged residues presumably present in the interior of the protein.

**Figure 4:**
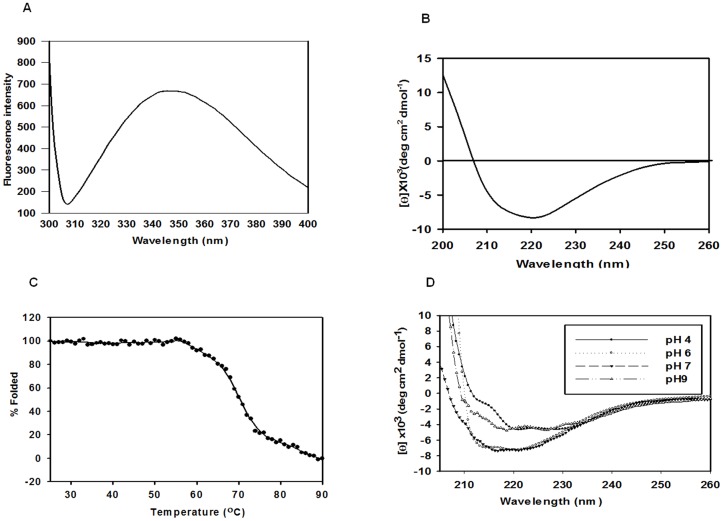
Conformation profiles of ADL as analysed by fluorescence and far-UV CD spectroscopy. (A) Intrinsic tryptophan fluorescence profile of *L. donovani* ADL shows emission maxima at 341 nm, indicating tryptophans are partially exposed. (B) Far-UV CD spectra (260 nm-200 nm) of *L. donovani* ADL protein shows ADL is comprised of mixture of α-helix and β-sheet. (C) Thermal denaturation curve (θ_222_) of *L. donovani* ADL showing co-operative unfolding with midpoint at ∼70°C. (D) Far-UV CD spectra of *L. donovani* ADL at pH range (4–9) showing maximum stability near to biological pH (7.0).

Primary sequence analysis of ADL shows three tryptophans at positions 4, 107 and 119. The fluorescence emission maxima of tryptophan are seen at 341 nm (Figure 4A). This indicates that these tryptophans are partially accessible to solvent. CD spectra (Figure 4B), shows that ADL has sufficient secondary structure elements 31% α-helix, 22% β-sheet, 47% random coil, as calculated using the K2D3 server (http://www.ogic.ca/projects/k2d3/) and is in agreement with the secondary structure compositions predicted by the PHD server (http://npsapbil.ibcp.fr) (Table 1).

**Table 1:** Comparison of secondary structure composition predicted by PHD server in members of SAM-decarboxylase superfamily with far-UV CD data of *L. donovani* ADL.

Organism	Protein	calculated/predicted by	α- helix	β-sheet	Random coil
*L. donovani*	ADL	Far-UV CD spectroscopy	31.4%	22.4%	47%
*L. donovani*	ADL	PHD server	30.30%	23.91%	45%
*L. infantum*	ADL	PHD server	27.2%	24%	58.4%
*L. major*	ADL	PHD server	27.2%	24.5%	48%
*H. sapiens*	AD	PHD server	24.5%	20%	54.9%
*S. tuberosum*	AD	PHD server	23%	24%	50%

### Stability analysis of *L. donovani* ADL as a function of temperature and pH

Since *Leishmania* has a digenetic lifecycle alternating between a promastigote stage in sandfly and an amastigote stage present in human macrophage between which it faces a variation in temperature (25°C in sand fly and 37°C in human macrophage), we analyzed the stability of *L. donovani* ADL over a range of temperature. The thermal denaturation studies show *L. donovani* ADL is stable up to 53°C, beyond which it begins to unfold. The T_m_ of protein was found to be ∼70°C and shows a sigmoidal curve of unfolding (Figure 4C), suggesting cooperative denaturation between native and denatured protein. Denaturation of this protein is a single-step process in which the protein undergoes a single transition from the native state to the denatured state. *L. donovani* ADL shows maximum stability at pH 7.0 (Figure 4D) and is sensitive to pH changes, resulting in heavy precipitation when pH is varied by 1.5. Unfolding studies with ANS shows that both urea and GdmCl denature through an intermediate species (figure S1, S2).

HYPERLINK “slot:sensitivity towards pH change as it ishows heavy precipitation even on 1.5 unit pH variation from biologicalchanges from the biologica l pH. The protein shows maximum stability at pH 7.0 which, corresponds to its biological pH. The protein shows maximum stability at biological pH (Figure 4D). Unfolding studies with ANS shows that both urea and GdmCl denatured the L. donovani ADL through an intermediate species (Figure S1, S2).”

### Substrate binding analysis of L. donovani ADL

**Figure 5:**
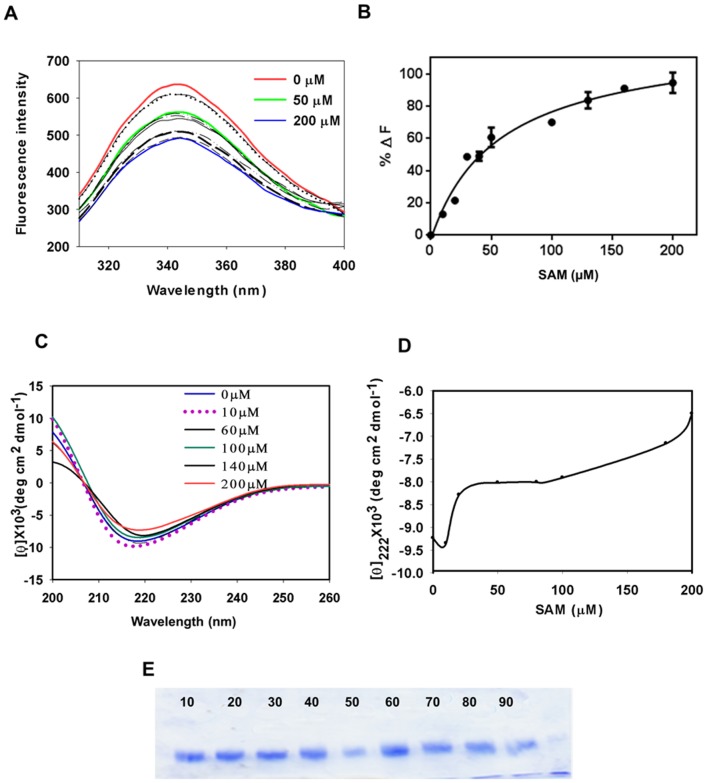
SAM binding analysis by fluorescence and far-UV CD spectroscopy. (A) Graph shows effect of increasing concentration of SAM causes quenching of tryptophan fluorescence (B) Saturation binding isotherm with dissociation constant 51 µM shows *L. donovani* ADL binds to SAM. (C) Far-UV CD spectra shows change in secondary structure with increasing concentration of SAM. (D) Change in secondary structure, with increasing concentration of SAM (0–200 µM) was monitored by molar ellipticity value at θ_222_, shows increasing concentration of SAM brings conformational change in protein. (E) Trypsin assisted limited proteolysis in presence of increasing concentration of SAM (10–80 µM) shows SAM binding does not affects folding pattern.

Despite lacking the essential Glu and Ser residues, *T. brucei* ADL has shown a significant effect on the catalytic activity of AD by playing an essential regulatory role [26], [27]. *L. donovani* ADL also lacks these residues and is possibly not involved in SAM decarboxylation. In order to determine whether *L. donovani* ADL binds with substrate while regulating the activity of AD as its *T. brucei* homolog, we examined its substrate binding capacity, using far-UV CD spectrum and tryptophan fluorescence. Tryptophan fluorescence with increasing concentrations of SAM shows quenching of the tryptophan emission maxima, suggesting SAM binding to the protein. A saturation isotherm was plotted for SAM and the binding of the ADL to SAM was identified by calculating the ΔF_max_ and K_d_ values from a fit saturation isotherm equation (Figure 5A and 5B), the ΔF_max_ and K_d_ values were found to be 194.5 and 51±5 µM. Far-UV CD spectrum suggests that increasing concentration of SAM induces a rearrangement of secondary structure causing a conformational change (Figure 5C and 5D). Taken together, the far-UV CD spectra and tryptophan fluorescence conclusively shows that *L. donovani* ADL binds SAM. To probe the effect of SAM on the structural organization of *L. donovani* ADL, a limited proteolysis experiment with trypsin was setup with SAM (0–80) µM. However, no digestion was seen (Figure 5E) indicating that SAM does not expose any trypsin cleavage site.

**Figure 6:**
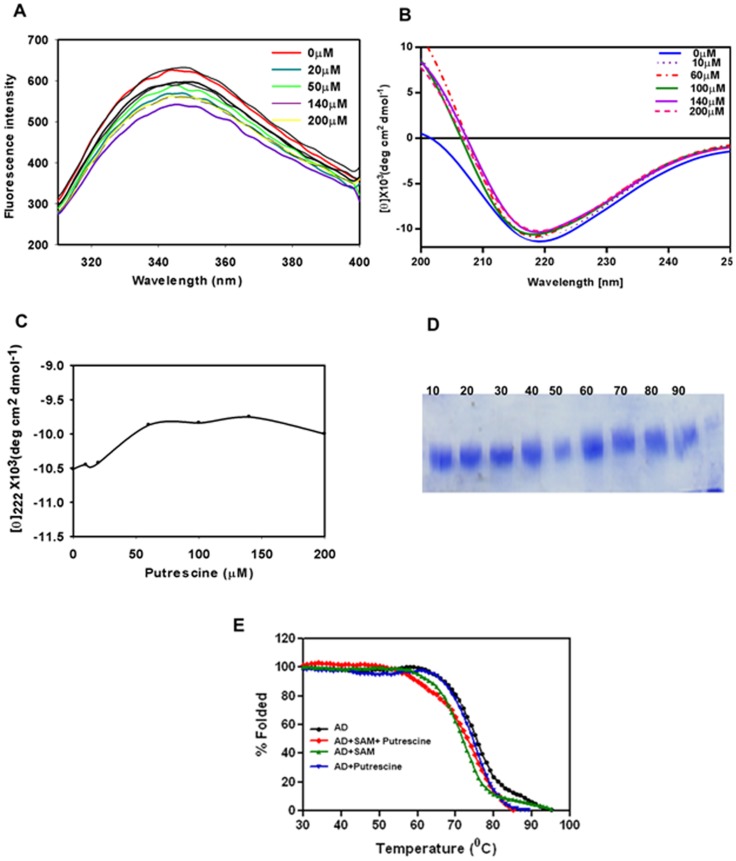
Putrescine binding analysis by fluorescence and far-UV CD spectroscopy. (A) Effect of increasing concentration of putrescine on tryptophan fluorescence shows putrescine quench *L. donovani* ADL in non-interpretable manner. (B) Far-UV CD spectra with increasing concentration of putrescine show change in secondary structure with increasing putrescine concentration. (C) Change in secondary structure, with increasing concentration of putrescine (0–200 µM) was monitored by molar ellipticity value at θ_222_, curve showing change in secondary structure with increasing concentration of putrescine upto 50 µM. (D) Limited proteolysis with increasing concentration of putrescine (10–90 µM) also does not show effect on folding pattern of *L. donovani* ADL. (E) Thermal denaturation profiles of native ADL (black), *L. donovani* ADL in complex with putrescine (blue), *L. donovani* ADL in complex with SAM (green) and *L. donovani* ADL in complex with both SAM and putrescine (red) reveals substrates binding causes decrease in thermal stability of *L. donovani* ADL.

Apart from SAM, putrescine also plays an essential role in the stability of *L. donovani* ADL, with the protein precipitation significantly reduced in a buffer containing putrescine during purification. Further, putrescine is also found in crystal structures of ADs from other organisms even when not being an explicit part of the buffer, indicating that AD actively binds putrescine [16]. It was reported in *T. cruzi* that the AD-ADL heterodimer complex needs putrescine for optimum activity while the corresponding *T. brucei* heterodimer complex is self sufficient to stimulate maximum activity and this difference has been correlated to their respective environments [26], [27]. However, the mechanism of putrescine stimulation and interaction in *T. cruzi* is still unknown. In *Leishmania* both promastigote and amastigote forms are capable of absorbing putrescine from the environment [51], [52]. In this context we analyzed the putrescine binding property of *L. donovani* ADL. Fluorescence spectroscopy shows quenching of tryptophan fluorescence in a non-interpretable manner with increasing concentration of putrescine which might be due to putrescine binding to multiple sites. The presence of multiple putrescine binding sites has also been reported earlier by Stanley *et al.*, 1994 [53]. Alternatively, putrescine being a cationic polyamine of small size, its electrostatic interactions with other charged amino acids surrounding tryptophan residues may also interfere with the tryptophan fluorescence (Figure 6A). The secondary structure content of *L. donovani* ADL show changes with increasing concentration of putrescine up to 50 µM (Figure 6B and 6C) and then remains constant, suggesting that the ADL binds putrescine as well. The observed change in secondary structure due to putrescine was less in comparison to SAM, probably due to its smaller size. Although this result shows that *L. donovani* ADL binds putrescine and get stabilized as observed in purification the detailed role and mechanism of putrescine is still not deciphered and requires further experimental work. In the absence of such, one can only conjecture that putrescine might either stimulate SAM binding activity of *L. donovani* ADL or bring conformational change that help in heterodimer complex formation with *L. donovani* AD. Increasing concentration of putrescine, like SAM, also has no effect on folding pattern of the ADL as seen in case of trypsin digestion in increasing concentration of putrescine (Figure 6D).

In far-UV CD thermal denaturation studies, it was also observed that the presence of SAM and putrescine decreases the T_m_ by 2–3°C as compared to the native protein (Figure 6E). It was already shown that *L. donovani* ADL binding to SAM and putrescine, resulting in conformational change in secondary structure of *L. donovani* ADL and this might be responsible for the decrease in T_m_.

### Homology modeling

To understand the structural rationale of protein ligand interactions, attempts were made to crystallize the protein, both in its *apo* form and with SAM and putrescine as ligands. Initial exploratory crystallization screens with a protein concentration of 4 mg/ml using standard crystal screens resulted in most of the drops showing heavy precipitation, with some drops precipitating immediately. To avoid precipitation, the protein concentration was lowered to 1–2 mg/ml and other parameters such as pH and temperature varied, which too did not result in diffracting crystals. In the absence of suitable crystals, computational model was generated to provide structural insights into the ligand binding aspects of *L. donovani* ADL. As mentioned earlier, PSI-BLAST did not result in any significant hits against PDB, so PHYRE fold search, was used for the identification of template with similar fold. PHYRE identified the human crystal structure, apo as well as in complex with ligand as having a similar fold with 100% confidence. The human AD (PDB ID 1MSV) which had the minimum E value was used as the template for homology modeling using Modeller 9.10 [42]. The homology models of ADs and ADLs of other *Leishmania spp.* and *Trypanosoma spp* were also generated using similar protocol. The output models were validated using standard tools and the models having minimum internal energy, with 92.7% residues in the favored region of the Ramachandran plot and an r.m.s.d value less than 2 Å was selected for further rationalization of protein ligand interaction and protein-protein interaction studies

**Figure 7:**
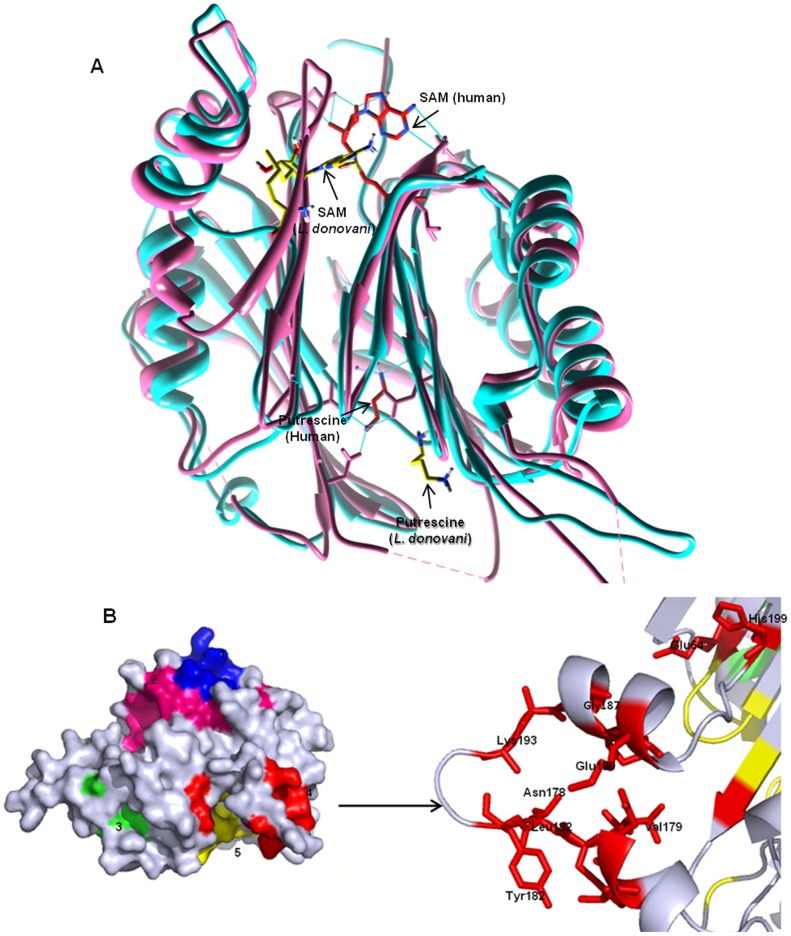
Homology modeling and prediction of SAM binding site. (A) Cartoon representation of the homology model of *L. donovani* ADL (cyan), superimposed on to the crystal structure of human AD (pink). Ligands observed in the human structure, SAMe (red) and putrescine (red) are also shown, as are the docked SAM and putrescine (yellow) to *L. donovani* ADL. Figure prepared with the help of Chimera 1.6.1. (B) Surface representation of the *L. donovani* ADL monomer showing the five binding pockets of SAM as predicted by Q-site prediction server. The different pockets are colored red, yellow, green, magenta and blue. The active site where SAM docked successfully is shown in inset (red). Figures are generated with the aid of Pymol molecular visualization tool [55].

The *L. donovani* ADL model is representative of the core architecture of proteins belonging to the SAM-decarboxylase superfamily, consisting of four layer αββα sandwich architecture with the β-sheets comprising of seven and eight β-strands arranged in antiparallel fashion (Figure 7A; Figure S3). This arrangement is slightly different from the human AD where each β-sheet comprises of eight strands. This is most likely due to *L. donovani* ADL having lesser number of residues as compared to human AD. The three tryptophan residues present in *L. donovani* ADL sequence are found partially exposed within the homology model, consistent with the intrinsic tryptophan fluorescence studies. The secondary structure composition of homology model is analogous to the predicted secondary structure composition with the proteins belonging to SAM decarboxylase superfamily and is consistent with the results obtained from far-UV CD spectroscopic studies.

### Active site architecture of *L. donovani* ADL

#### (i) SAM binding

**Figure 8:**
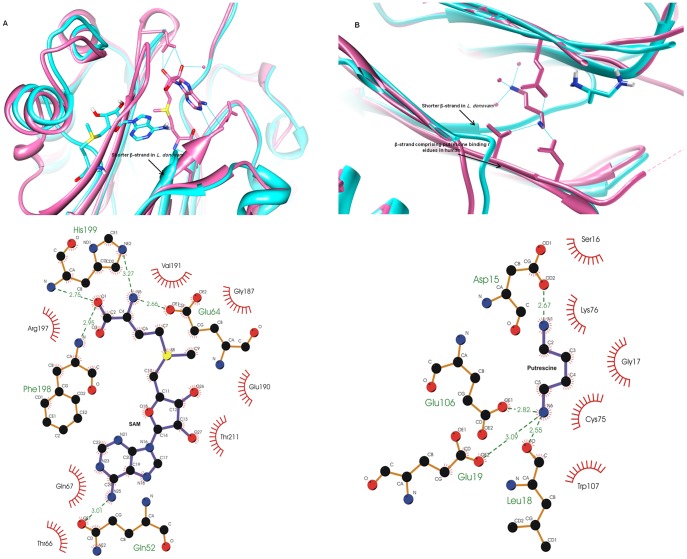
Binding site of SAM and putrescine obtained by molecular docking. (A) Cartoon representation of homology model of *L. donovani* ADL (cyan) with the docked SAM (cyan), superimposed on SAM-bound crystal structure of human AD (pink) illustrating the differences in relative SAM binding positions. LIGPLOT diagram showing the interactions of the docked SAM with the ADL residues are shown in the bottom panel (B)Cartoon representaton of putrescine (cyan) docked to the ADL homology model. For comparison, the corresponding region of the human AD structure is also shown, with its putrescine (pink). The bottom panel shows LIGPLOT representation of the interaction between putrescine and ADL.

Having seen that *L. donovani* ADL binds SAM and putrescine, we tried to locate the binding sites from the homology model. However, multiple sequence alignment (Figure 2) was not able to provide any relevant information regarding the active site architecture of *L. donovani* ADL, as most of the functional residues are different in *L. donovani* ADL. So, in order to probe the active site architecture we used the Q-site finder server for active site prediction. Q-site finder gave five probable binding pockets for SAM (colored differently in Figure 7B) and these were taken as the starting grids for docking the ligand using AUTODOCK 3.0. However, in four of these sites SAM could not be docked favorably. The energetically favorable (docking energy −10.79 kcal/mol) binding pocket for SAM, confirmed by docking, shown in red color in inset (Figure 7B), was considered for further analysis. Comparison of the SAM binding with human AD crystal structure [50] showed significant differences (Figure 8A and Table 2). In the human AD, SAM lies at the edge of the β-sheet interface, interacting with the residues belonging to both sheets while in *L. donovani* ADL, the docked SAM lies near one β-sheet with its adenine ring in the β interface and the methionine tail extending to the α-β interface. This difference is most likely caused by the shorter length of β-strand (Leu60-Met65 in *L. donovani* ADL and Gln60-Ser66 in human AD) which is involved in interaction with the SAM in human AD. Closer examination of the *L. donovani* ADL docked with SAM with the crystal structure of human AD reveals the differences in the ligand binding (Table 2). Glu67 and Glu247 are involved in binding SAM in human AD while in *L. donovani* ADL, SAM interacts with only one similar residue Glu64 with additional interactions from Gln52, Phe198 and His199.

**Table 2:** Showing H-bond interaction of SAM with residues of *L. donovani* ADL and their comparsion with SAM bound crystal structure of human AD.

SAM binding residues in *L. donovani* and human	Residues	H-bond	Bond length (Å)
*L.donovani* ADL	Gln52	**OE1…N6**	3.00
	His199	**N…OXT**	2.75
	His199	**NE2…N**	3.27
	Glu64	**OE1…N** **N…OXT**	2.66
	Phe198		2.95
AD Human	Glu 67	**N…N1**	2.96
		**O…N6**	2.95
	Glu 247	**OE1…O3**	2.82
		**OE2…O2**	2.86

Comparative analysis also shows that in *L. donovani* ADL, the adenine ring of SAM is stabilized by only one hydrogen bond, through the interaction of its amino group with the α-carboxyl oxygen of Gln52 which is different from human AD [50]. *L. donovani* ADL does not have any interaction with ribose sugar unlike human AD. However, this is adequately compensated by the carboxylic tail of SAM methionine being stabilized by the α-amino group of Phe198 and the amide nitrogen of His199. The terminal amino group of SAM is stabilized by H-bond interaction with nitrogen of His199 imidazole ring and oxygen atom of side chain carboxylate of Glu64 (Figure 8A; Table 2).

As can be seen, the residues involved in SAM binding are completely different from the human AD. Multiple sequence alignment based comparison of residues involved in SAM binding in *L. donovani* ADL with ADLs and ADs of trypanosomatids and human AD shows that the residues found to be involved in SAM binding with *L. donovani* ADL are conserved in *Leishmania* ADLs but are different even from ADL of *Trypanosoma spp.*, ADs of all trypanosomatids and human AD which indicates the probability of novel mode of mechanism of group of ADLs in *Leishmania spp.* (Figure 2).

#### (ii) Putrescine binding site

The putrescine binding site of AD from known organisms were studied in order to know the residues which are involved in putrescine binding in case of *L. donovani* ADL. This site is comprised of adjacently located negatively charged residues. The homology model of *L. donovani* ADL was screened for such sites and docking was done by including glutamate residues with negatively charged micro-environment in the grid. Four putrescine binding sites were observed in the model of *L. donovani* ADL with docking energy. −5.30 to −6.36 consistent with the experimental observation of putrescine binding to multiple sites. On comparison of the docked putrescine-ADL with the corresponding human AD crystal structure (PDB ID 1I7B) [50], one of the four binding site was found to be identical to one putrescine binding site of human AD (Figure 8B). Comparison of other putrescine binding site shows that the docked putrescine adopts a different position in *L. donovani* ADL, due to the short length of β-strand and completely different orientation of proceeding loop as compared to the human AD. The putrescine binding site of human AD interacts with the residues from both β-sheets while in *L. donovani* ADL putrescine is closer to one β-sheet and interacts with the residues on this sheet only. The putrescine binding pocket of *L. donovani* is comprised of similar Glu and Asp residues as found in the putrescine bound crystal structure of human AD, the only difference being the presence of Leu in place of Thr (Table 3). The other three putresceine binding sites, obtained in *L. donovani* ADL by docking studies also comprised of similar residues.

**Table 3:** Showing interaction of putrescine with residues in ADL and their comparison with interaction involved in putrescine binding in human AD.

Protein	Residues	H-bond	Bond length (Å)
*L. donovani* ADL	Asp15	**OD2…N2**	2.67
	Leu18	**O…N1**	2.55
	Glu19	**OE2…N1**	3.09
	Glu106	**OE1…N1**	2.82
Human AD	Glu15	**OE2…N1**	2.69
	Asp174	**OD2…N1**	2.85
	Thr176	**OG1…N1**	2.90

Binding residues analysis of SAM and putrescine shows that SAM binding site of *L. donovani* ADL is sufficiently different from *L. donovani* and human AD. However, in case of putrescine, binding pattern and sequence alignment shows that binding residues are similar in *L. donovani* ADL and human AD. Based on the SAM and putrescine binding studies of L. donovani ADL, it can be concluded the mode of SAM binding is probably the major difference with human AD that needs to be exploited in any novel inhibitor designing.

### Interaction analysis of ADL from *Leishmania spp.*

**Figure 9:**
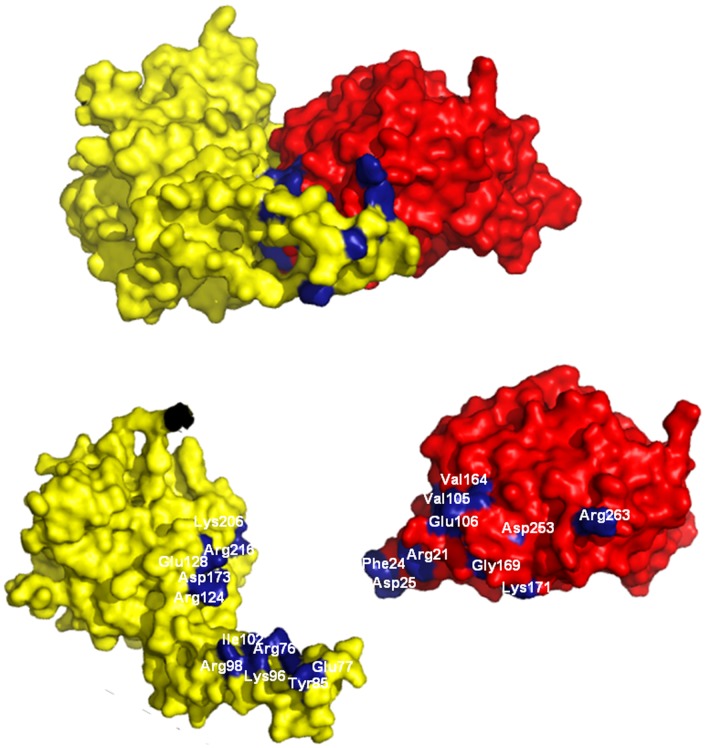
Surface representation of AD:ADL heterodimer, as obtained by ClusPro 2.0. Surface representation of the *L. donovani* AD-ADL heterodimer complex with AD (yellow) and ADL (red). Both monomers and the residues involved in heterodimer complex formation are shown below. The interaction is stabilized by salt bridges between Lys96, Arg124, Asp173, Lys206 and Arg216 of *L. donovani* AD with Asp253, Glu106, Arg21, Asp25 and Asp25 of *L. donovani* ADL.

As AD and ADL in *Trypanosoma* have been shown to interact together to form the catalytically active heterodimer complex, wesought to see if this heterodimer formation is possible in *Leishmania* as well. We took recourse to computational methods, employing the STRING 9.0 and ClusPro 2.0 softwares for this purpose. The interaction studies of homology models of ADL, from members of trypanosomatids superfamily, with their corresponding AD shows interaction, according to STRING 9.0. Further, as a negative control, a pair of non-interacting proteins, *L. donovani* nucleotide diphosphatase kinase b and gamma-glutamylcysteine synthetase were input to the STRING 9.0 and no interaction was observed (Data not shown). Further, ClusPro 2.0 and GRAMM-X shows positive docking result for *L. donovani* as well as *T. brucei* AD-ADL pair. From these results, it is reasonable to expect that these two proteins do interact as a heterodimer for its function, as in *Trypanosoma*. Analysis of the docked structures shows that the interaction is stabilized by salt bridges between Lys96, Arg124, Asp173, Lys206 and Arg216 of AD with Asp253, Glu106, Arg21, Asp25 and Asp25 of *L. donovani* ADL (Figure 9). All interacting residues in the two proteins along with their interaction are shown in Figure 9 and the interaction score of all the servers are summarized in Table 4. These computational results strongly indicate that AD and ADL do have the potential to form a heterodimer, as in trypanosoma. We have recently cloned, overexpressed and purified *L. donovani* AD (data not shown). Preliminary interaction analysis using pull-down assays suggest that t that L. donovani AD indeed form a heterodimer complex with ADL (Figure S4). Encouraged with this result, further characterization of the complex has also been initiated..

**Table 4:** Showing AD-ADL interaction in trypanosomatids and their respective interaction score.

Interacting proteins	String 9 score	GRAMM X result	ClusPro 2 weighed score
*L. donovani* AD-ADL	0.899	Positive	−1279.4
*L. infantum* AD-ADL	0.899	Positive	−1189.1
*L. major* AD-ADL	0.899	Positive	−1279.4
*L. brazilensis* AD-ADL	0.899	Positive	−1165.4
*T. cruzi* AD-ADL	0.899	Positive	−1274.6
*T. brucei* AD-ADL	0.899	Positive	−1214.4

## Conclusion

Trypanosomatids including *L. donovani* have two copies of the protein belonging to the S-adenosylmethionine decarboxylase superfamily, annotated here as AD and ADL. While AD from different sources has been structurally and functionally characterized, not much is known about ADL, although in *Trypanosoma* it plays an essential role. To better understand this protein, we have cloned expressed and purified *L. donovani* ADL in its native conformation. *L. donovani* ADL is stable even in the absence of *L. donovani* AD, unlike *Trypanosoma* where ADL shows partial aggregation in absence of AD. We carried out its biochemical, biophysical and computational characterization to analyze its structural and functional properties. Based on this study, *L. donovani* ADL is a member of group comprising novel proteins of S-adenosylmethionine decarboxylase superfamily i.e, ADLs, sharing some key features of this superfamily such as having similar secondary structural components, tertiary structure and a dimeric quaternary association of native protein. Besides these common features, leishmanial ADL also exhibits several distinct features from human AD and even from ADs of trypanosomatids. It has no significant sequence identity with the other members of the SAM decarboxylase superfamily, does not undergo autoprocessing reaction and should therefore not be annotated as proenzyme. Interestingly, our study has also shown that *L. donovani* ADL binds to SAM and putrescine, natural substrates of AD. To rationalize the ligand binding, computational homology modeling was undertaken followed by docking of these ligands. Homology modeling reveals that the tertiary structure exhibits the classical αββα sandwich arrangement albeit with a subtle difference. Instead of the predominant arrangement of 8 β-strands in each β-sheet, *L. donovani* ADL appears to have a 7-stranded and an 8-stranded sheet. Docking studies confirmed that ADL could bind SAM and putrescine. On comparison with the crystal structure of human AD, the SAM binding residues are distinctly different. *L. donovani* ADL is also involved in interaction with AD favoring heterodimer complex formation as in *Trypanosoma*. The fact that ADL binds to SAM and putrescine suggests that the functioning of the AD-ADL heterodimer might be significantly different from *Trypanosoma*. Two possible mechanisms are plausible: Both AD and ADL can be equally active during catalysis and it may be that ADL might still play only a limited regulatory role and the actual mechanism needs to be delineated experimentally. In either case, it can be said that ADL, as compared to AD, appears to be a better candidate as a potential drug target. However, further characterization and validation of this complex is necessary. To this end, we have initiated purification of *L. donovani* AD as well and preliminary results suggest that L. donovani AD indeed form a heterodimer complex with ADL. Further analysis of stability of this heterodimer complex is in progress.

## Supporting Information

Figure S1 **Cartoon representation of homology model of monomeric** ***L. donovani*** **ADL (cyan), superimposed with structure of human AD (pink), shows that** ***L. donovani*** **ADL also have same structural organization αββα as in case of human AD, but ADL has one β-strand missing due to short length as shown in figure.** Figure is made with the help of Chimera 1.6.1.
(TIF)

Figure S2 **Unfolding studies of ADL in presence of urea.** The changes in tertiary structure were monitored by fluorescence studies using tryptophan as intrinsic fluorophore and ANS as extrinsic fluorophore. (A–B) Effect of increasing concentration of urea on tryptophan fluorescence was monitored at tryptophan emission maxima 341 nm, shows increasing concentration of urea causes gradual red shift of ADL due to unfolding of protein with complete unfolding at 6 M urea. (C–D) ANS fluorescence emission spectra with increasing concentration of urea, monitored at 465 nm. Graph shows emission maxima increases with increase in concentration of urea up to 0.5 M urea, then gradually decreased with minima at 6 M urea, due to loss of hydrophobic patches.
(TIF)

Figure S3 **Unfolding studies of** ***L. donovani*** **ADL in presence of GdmCl.** The changes in tertiary structure were monitored by fluorescence studies using tryptophan as intrinsic fluorophore and ANS as extrinsic fluorophore. (A–B) Effect of GdmCl on tryptophan fluorescence of *L. donovani* ADL monitored at 341 nm emission maxima. Graph shows increased concentration of GdmCl causes unfolding of protein with maximum transition at 1.5 M GdmCl and protein gets fully exposed at 2 M concentration. (C–D) ANS fluorescence emission spectra with increasing concentration of GdmCl monitored at 465 nm shows emission maxima increases with increasing concentration of GdmCl up to 0.5 M GdmCl, and then gradually decreased to a minimum value at 4 M GdmCl.
(TIF)

Figure S4 **AD-ADL interaction observed from GST pull down assay.** The cell lysate containing AD-GST construct (in Tris-HCl pH 7.5 and 150 mM NaCL) was incubated with glutathione agarose for two hours and the unbound cell lysate discarded and washed with buffer containing Tris-HCl pH 7.5and 1M NaCl, before incubating with cell lysate containing 6×His-ADL for 2 hours, washed with 5 column volumes of the same buffer and eluted with reduced glutathione. A similar experiment using GST alone instead of AD-GST was also performed as control. The elution products were analyzed on 12% SDS PAGE (top panel): Lane 1 marker, Lane 2 elution of GST with ADL, Lane 3: elution of AD-GST with ADL and Lane 4: AD alone. The band at ∼60 kDa corresponds to full length AD-GST while the band at ∼35 kDa corresponds to the autocatalyzed N-terminal fragment of AD (9 kDa) fused to 26 kDa GST (Mr 35 kDa) as well as ADL (Mr 33 kDa). To resolve this, the eluted products were then probed with anti-GST (middle panel) and anti-His (bottom panel) antibodies which show the presence of the two species. The absence of a band corresponding to ADL in Lane 2 in the anti-His blot confirms specific AD: ADL interaction.
(TIF)
