# Supplementary figures and images for: Characterization of a Novel Putative S-Adenosylmethionine Decarboxylase-Like Protein from Leishmania donovani
Source: PLoS One. 2013 Jun 19;8(6):e65912. doi: 10.1371/journal.pone.0065912 (PMC3686867; doi:10.1371/journal.pone.0065912)

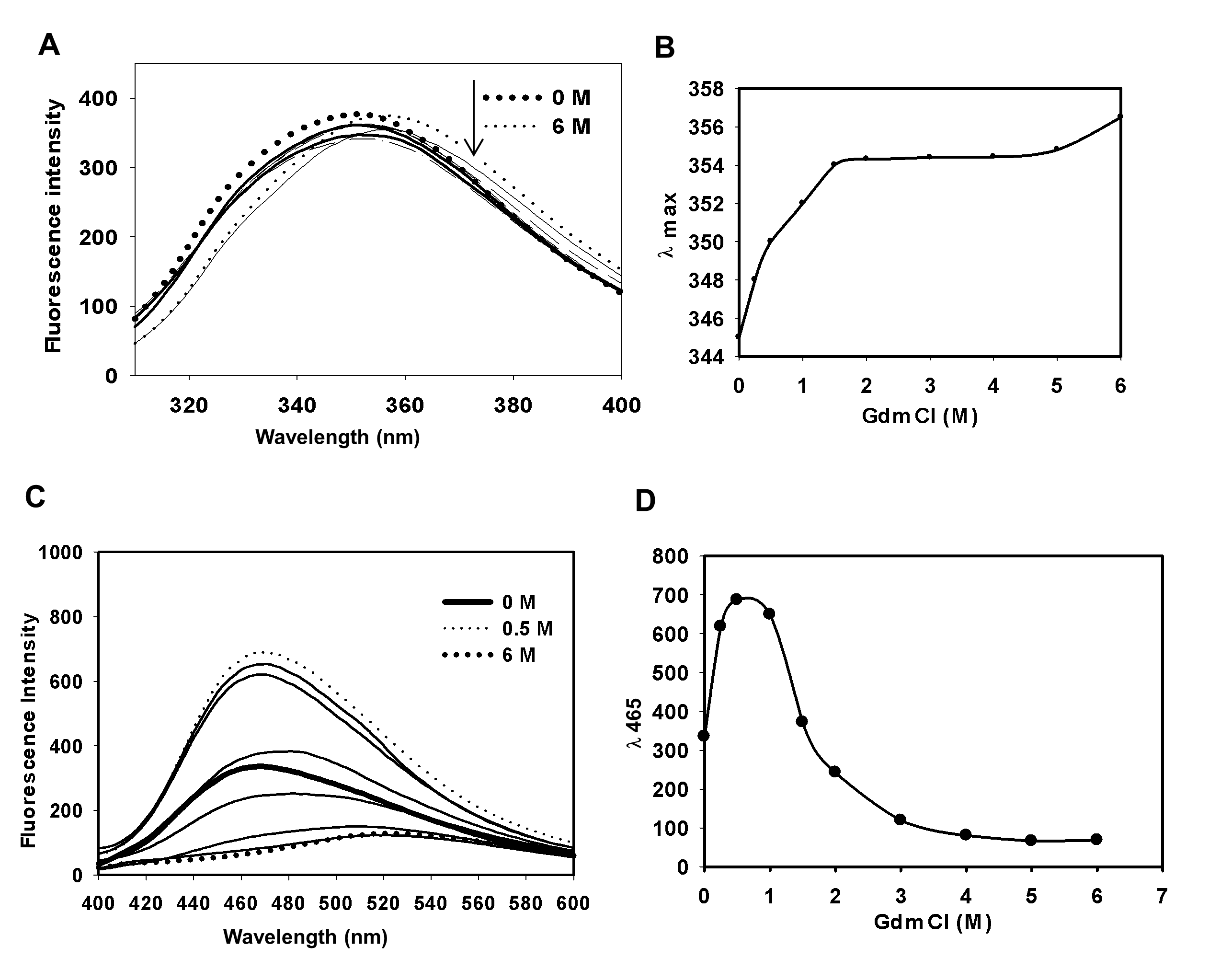

Supplement: Figure S1 — Cartoon representation of homology model of monomeric L. donovani ADL (cyan), superimposed with structure of human AD (pink), shows that L. donovani ADL also have same structural organization αββα as in case of human AD, but ADL has one β-strand missing due to short length as shown in figure. Figure is made with the help of Chimera 1.6.1. (TIF) [file pone.0065912.s001.tif]

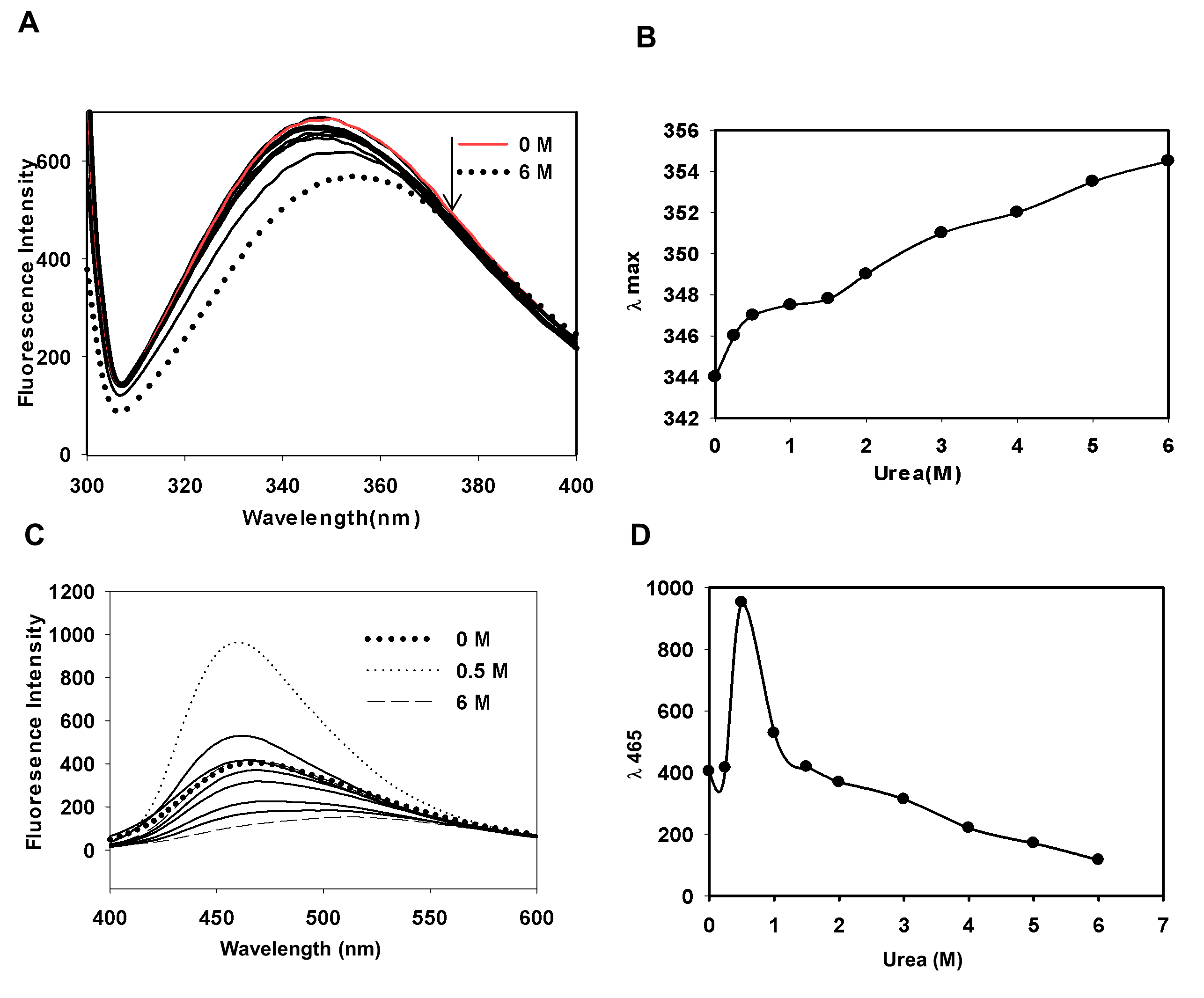

Supplement: Figure S2 — Unfolding studies of ADL in presence of urea. The changes in tertiary structure were monitored by fluorescence studies using tryptophan as intrinsic fluorophore and ANS as extrinsic fluorophore. (A–B) Effect of increasing concentration of urea on tryptophan fluorescence was monitored at tryptophan emission maxima 341 nm, shows increasing concentration of urea causes gradual red shift of ADL due to unfolding of protein with complete unfolding at 6 M urea. (C–D) ANS fluorescence emission spectra with increasing concentration of urea, monitored at 465 nm. Graph shows emission maxima increases with increase in concentration of urea up to 0.5 M urea, then gradually decreased with minima at 6 M urea, due to loss of hydrophobic patches. (TIF) [file pone.0065912.s002.tif]

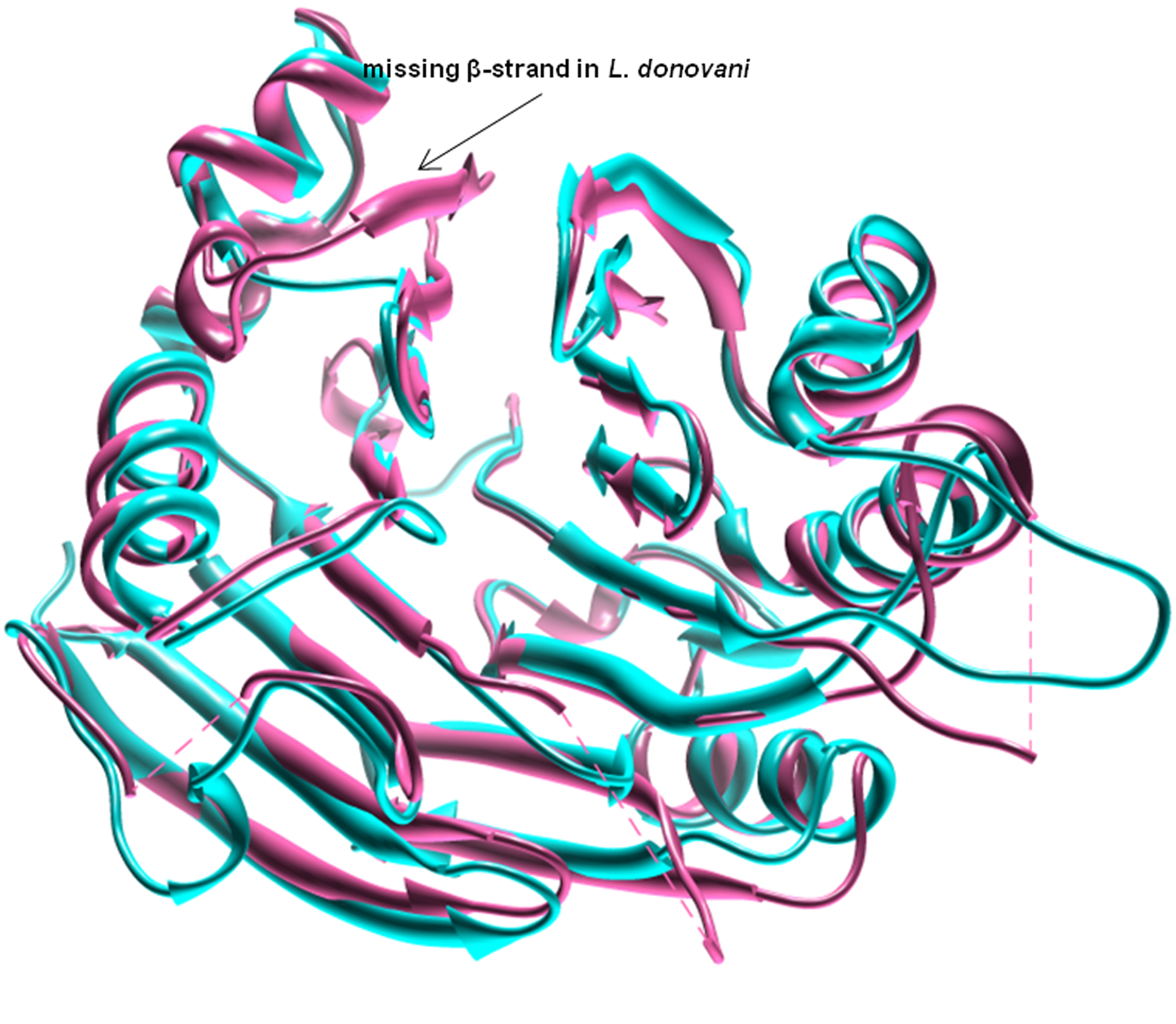

Supplement: Figure S3 — Unfolding studies of L. donovani ADL in presence of GdmCl. The changes in tertiary structure were monitored by fluorescence studies using tryptophan as intrinsic fluorophore and ANS as extrinsic fluorophore. (A–B) Effect of GdmCl on tryptophan fluorescence of L. donovani ADL monitored at 341 nm emission maxima. Graph shows increased concentration of GdmCl causes unfolding of protein with maximum transition at 1.5 M GdmCl and protein gets fully exposed at 2 M concentration. (C–D) ANS fluorescence emission spectra with increasing concentration of GdmCl monitored at 465 nm shows emission maxima increases with increasing concentration of GdmCl up to 0.5 M GdmCl, and then gradually decreased to a minimum value at 4 M GdmCl. (TIF) [file pone.0065912.s003.tif]

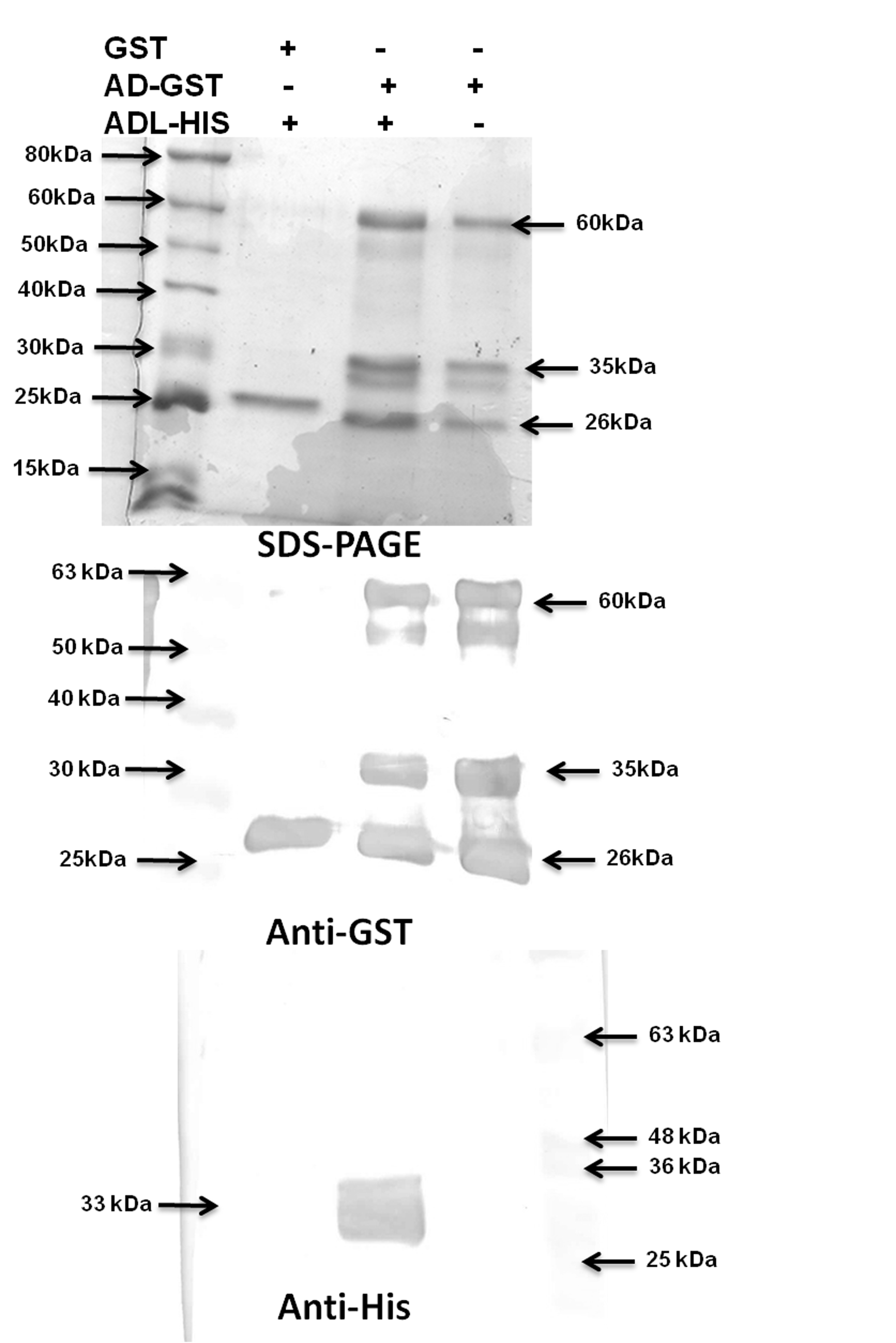

Supplement: Figure S4 — AD-ADL interaction observed from GST pull down assay. The cell lysate containing AD-GST construct (in Tris-HCl pH 7.5 and 150 mM NaCL) was incubated with glutathione agarose for two hours and the unbound cell lysate discarded and washed with buffer containing Tris-HCl pH 7.5and 1M NaCl, before incubating with cell lysate containing 6×His-ADL for 2 hours, washed with 5 column volumes of the same buffer and eluted with reduced glutathione. A similar experiment using GST alone instead of AD-GST was also performed as control. The elution products were analyzed on 12% SDS PAGE (top panel): Lane 1 marker, Lane 2 elution of GST with ADL, Lane 3: elution of AD-GST with ADL and Lane 4: AD alone. The band at ∼60 kDa corresponds to full length AD-GST while the band at ∼35 kDa corresponds to the autocatalyzed N-terminal fragment of AD (9 kDa) fused to 26 kDa GST (Mr 35 kDa) as well as ADL (Mr 33 kDa). To resolve this, the eluted products were then probed with anti-GST (middle panel) and anti-His (bottom panel) antibodies which show the presence of the two species. The absence of a band corresponding to ADL in Lane 2 in the anti-His blot confirms specific AD: ADL interaction. (TIF) [file pone.0065912.s004.tif]
